# Cardiac Anomalies Associated with Funnel Chest

**Published:** 1947

**Authors:** G. E. F. Sutton

**Affiliations:** Physician to the Bristol Royal Hospital


### CARDIAC ANOMALIES ASSOCIATED WITH FUNNEL CHEST

BY

G. E. F. SUTTON, *M.C.*, M.D., M.R.C.P.

*Physician to the Bristol Royal Hospital*

EVERY practitioner has met with patients who have manifested varying degrees of funnel chest, and in view of the frequency of subjective symptoms, together with systolic murmurs and other cardiac anomalies in this condition, the question of organic heart disease is sure to have arisen with regard to some particular case. The literature on this subject shews conflict of opinion between various authorities. Dr. A. Lincoln Brown[1] defines Pectus Excavatum as :

" That deformity of the thoracic cage which is characterized by a funnel-shaped depression of the sternum, the apex of which is located at approximately the level of the junction of the xiphoid with the body." He quotes the report on a patient which was written in Latin by J. Brauhin in 1600. I have translated this somewhat freely as follows :

" Congenital internal bending back of the sternum and ribs as the cause of difficulty in breathing.

" My father showed me a boy seven years old at the house of a nobleman of Andlau, whose ensiform cartilage and other parts of the chest bone, together with the ribs, was bent back towards the internal parts, so that a great hollow appeared there. The diaphragm, he pointed out, seemed to contract those parts to a great extent. Right from his birth this boy had laboured under this serious physical drawback, breathing with difficulty and having an obnoxious cough. When we saw him he was very seriously ill, so much so that suffocation seemed imminent, and a viscid sputum was being brought up. The lady of Andlau said that she had a nephew younger than the other boy who suffered from the same condition and whose life was in similar danger."

Brown states that the heart tends to be fixed near the midline by the diaphragmatic attachment of the pericardium, but it may be displaced either to the left or right ; though he is of the opinion that the greatest cardiac embarrassment is caused when the heart is fixed near the midline and is thus directly compressed by the depressed sternum. He gives these theories of causation : (1) Intra-uterine pressure, as from a knee or elbow pressing on the foetal sternum ; (2) Intra-uterine overdevelopment of the corpus sterni ; (3) Arrested development of the sternum ; (4) Rhachitic influence on the development of the sternum and the entire thoracic cage ; (5) Obstruction of the air passages, especially by tonsils and adenoids ; (6) Genetic or ; (7) Neurological factors.

He dismisses the intra-uterine theories as fantastic, and considers that funnel chest has never been recorded in an authenticated case of rickets, and further that tonsils and adenoids are unlikely causes as funnel chest is so unusual in such cases. Genetic factors are the most important, and he quotes Nowak who found that out of 30,000 Viennese school-children, twelve (0.04 per cent.) were afflicted with this condition and all had had indications of it since birth. Out of one hundred and six individuals of the families who were examined, forty-one had it (38 per cent.) : twenty-four males and seventeen females. Brown then records two cases both of which were greatly relieved by operative measures. One had a systolic murmur at the apex : the other had a loud pulmonary systolic murmur on expiration as well as a split pulmonary second sound, and complained of dyspnoea on exertion. The family history in this case revealed that the father, brother and sister also had a less degree of funnel chest. Brown states that the release of diaphragmatic attachments to the medial aspect of the sternum was the greatest single factor in the correction of the deformity. He gives the indications for the operation as follows : (*a*) prophylactic ; (*b*) cosmetic in women ; (*c*) when embarrassment in one form or another results from the condition.

Richard H. Sweet[2] records successful operations on two sisters. The older, a girl of fourteen, had dorsal scoliosis as well as marked funnel chest, and she suffered from severe attacks of paroxysmal tachycardia : there was a harsh systolic murmur at the apex and the pulmonary second sound was split. The younger girl aged five also had some scoliosis in addition to the funnel chest : in her case there was a double mitral murmur of moderate intensity as well as a loud systolic murmur heard all over the praecordium, which was accompanied by a thrill, and became louder as it was traced to the left to reach a maximum intensity under the angle of the scapula.

In describing the case of a healthy twenty-six year old airman with marked funnel chest, Manning[3] again records an apical systolic murmur. He states that severe deformities of the spine tend to result in pulmono-cardiac failure. In support of this statement he quotes Kerwin who concluded that " The significant pathological lesions are the result of pulmonary hypertension which eventually leads to right-sided heart failure, and appears to be due to mechanical interference with respiratory function."

On the other hand, William Evans[4] records sixteen cases, all of which had been referred to the cardiac department of the London Hospital because of the suspicion of heart disease. In every patient a systolic murmur was present, but in none was there any evidence of heart failure. He suggests that the murmur might be produced by the impact of the heart on the sternal bulge. He states further that X-ray reports have been misleading in that they have failed to distinguish between true and what was only an apparent enlargement brought about by the displacement. He insists that the symptoms were never the result of heart enlargement or embarrassment, but were the direct outcome of the physical inactivity or mental anxiety, resulting from the unwarranted invalidism. He quotes Lang as having found depression of the sternum in 3.5 per cent. of children in the first year of schooling. It is interesting to compare the evolutionary theories which he records, and also to contrast the opinion which he deduces, with that of Brown as given above : (1) Abnormal length of the ribs (Flesch,

1873) ; (2) Occupational factors, *e.g.* cobblers (Laënnec, 1819) ; (3) Arrested development of the sternum (Ebstein, 1882) ; (4) Abnormal postural development in a child of slender build arising from faulty posture (Kuhns, 1931).

Evans states that in his cases there was no evidence that they had resulted from congenital maldevelopment and in all it appeared in early childhood, when it might have been the outcome of a disturbance of the mechanism of respiration.

Although Carey Coombs did not mention funnel chest, he recorded four cases of fatal cardiac failure occurring in persons with angular deformity of the spine. Autopsy was performed on two of these patients and no other lesion was found. In his commentary he points out that in every case cardiac symptoms ensued shortly before death. Three influences, he says, are blamed for the heart failure : (1) Aortic kinking ; (2) Inadequate air space in the lungs, and (3) Restriction of the diaphragmatic excursion. "Whatever the explanation," he concludes, "it is clear that there is a real danger of untimely death from cardiac failure in the victims of angular deformity of the spine ".

As I had not then read Carey Coombs's[5] report, I was frankly puzzled when I was asked to see a patient in consultation with a doctor about fourteen years ago. This patient, a female aged thirty, had had severe funnel chest and scoliosis as long as she could remember, yet she had remained well until the last few months when she had complained of increasing dyspnoea and palpitation. There was obvious severe heart failure with marked dyspnoea. The heartbeat was regular at the rate of 140 and a soft systolic murmur was present at the apex. At that time I could only suggest to the experienced practitioner, who was equally puzzled, that in some way the deformity of chest and spine had caused the heart failure which was clearly present. The patient died within a few days and autopsy revealed only a dilated heart with no evidence of valvular or myocardial changes.

In November, 1946, a young man was referred to me as a case of heart disease. He had been rejected by a National Service Board in 1940, because of heart disease though he himself felt quite well. On examination, in addition to an apical systolic murmur I found that there was some apparent diffusion of the impulse caused by a moderate degree of funnel chest. The man was nervous but there was no evidence of thyrotoxicosis. My report to his doctor stated that "This type of murmur has been described in association with funnel chest when no organic heart disease is present. I have known it to be diagnosed as mitral disease and also to be queried as bacterial endocarditis because a murmur is discovered whenever the patient has a temperature from influenza or some other cause. In my opinion the man should be regarded as a normal life, for none of the signs and symptoms of organic heart disease is present." X-ray and cardiographic examinations were subsequently negative.

It seems clear from the literature that in the majority of cases funnel chest is not associated with heart failure and that heart disease is frequently erroneously diagnosed because of a systolic murmur, or the presence of other signs and symptoms. Nevertheless, when a severe degree of funnel chest is present, and especially if this be combined with deformity of the spine, symptoms of severe heart failure may ensue rather suddenly. In such cases there seems to be a tendency to marked tachycardia which does not readily respond to treatment.

### REFERENCES.

[1] A. Lincoln Brown, *J. Thoracic Surg.*, 1939, ix. 164.

[2] Richard H. Sweet, *Ann. Surg.*, 1944, cxix. 922.

[3] G. W. Manning, *Canad. M. A. J.*, 1945, liii. 550.

[4] W. Evans, *Brit. Heart J.*, 1946, viii. 162.

[5] Carey F. Coombs, *Brit. J. Surg.*, 1930–31, xviii. 326.

